# Bile duct ligation-induced cirrhosis does not alter the blood-brain barrier permeability to sucrose in rats

**DOI:** 10.1007/s11011-024-01486-6

**Published:** 2024-12-05

**Authors:** Mohammad K. Miah, Ulrich Bickel, Reza Mehvar

**Affiliations:** 1 Department of Pharmaceutical Sciences, Texas Tech University Health Sciences Center, Amarillo, Texas USA; 2 Clinical Pharmacology & Quantitative Pharmacology, CPSS, AstraZeneca, Boston, Massachusetts USA; 3 Center for Blood-Brain Barrier Research, Texas Tech University Health Sciences Center, Amarillo, Texas USA; 4 Department of Biomedical and Pharmaceutical Sciences, School of Pharmacy, Chapman University, 9401 Jeronimo Road, Irvine, California USA

**Keywords:** Blood-brain barrier permeability, Liver cirrhosis, Cholestasis, Bile duct ligation, [^13^C]Sucrose, Apparent brain uptake clearance

## Abstract

Contradictory results have been reported about the effects of liver diseases on the blood-brain barrier (BBB) permeability to markers. For instance, both an increase and no change in the BBB permeability to BBB markers sodium fluorescein and Evans blue have been reported in experimental cholestasis induced by bile duct ligation (BDL) in rats. These contradictory effects might be due to inherent limitations of these markers and/or methodological issues. Here, we investigated the time course of the impact of BDL in rats on BBB permeability using a recently developed stable isotope labeled marker [^13^C]sucrose, which is expected to be devoid of limitations of other markers, such as sodium fluorescein. At various times (five days, two weeks, and four weeks) after BDL or sham surgery, the brain uptake clearance (*K*_*in*_) of [^13^C]sucrose was estimated using quantitation of the marker in plasma, blood, and brain by a specific LC-MS/MS analytical method. BDL caused substantial increases in the plasma concentrations of liver biochemical markers (bilirubin, total bile acids, ammonia, and cholesterol) and reduced liver cytochrome P450 content and metabolic activities. However, compared with the sham group, the plasma or blood AUC, brain concentrations, and *K*_*in*_ of [^13^C]sucrose in BDL animals remained unchanged at all the studied times. Additionally, we observed a negative correlation between the sucrose *K*_*in*_ and plasma total bile acids concentrations in the BDL animals. It is concluded that cholestatic liver disease, induced by BDL surgery in rats, does not significantly affect the BBB permeability to sucrose up to 4 weeks after the surgery.

**Supplementary Information:**

The online version contains supplementary material available at 10.1007/s11011-024-01486-6.

## Introduction

Liver diseases are known to negatively affect the functions of remote organs, including the brain (D'Mello and Swain 2011). Various reports have indicated that liver diseases may lead to cognitive impairments and, in severe cases, to hepatic encephalopathy and coma (Butterworth 2013; Claeys et al. 2021; Ohikere and Wong 2024; Prasad et al. 2007). Despite some controversy (Butterworth 2013; Nguyen 2012), it is generally believed that alterations in the blood-brain barrier (BBB) structure and/or function are one of the mechanisms of encephalopathy and coma associated with acute liver failure (Butterworth 2016; Cauli et al. 2011; Chastre et al. 2013; Chen et al. 2009, 2013; McMillin et al. 2015). In addition to acute liver failure, chronic liver diseases, such as cirrhosis, may also be associated with neurological deficits and cognitive decline, potentially leading to hepatic encephalopathy at later stages (Claeys et al. 2021). However, compared with acute liver failure, the effects of chronic liver diseases on BBB permeability are less understood.

Bile duct-ligated (BDL) rats have been extensively used as a reliable animal model of cholestasis-induced liver injury, resulting in portal inflammation, fibrosis, and secondary biliary cirrhosis (Liu et al. 2013). Additionally, BDL rats have been used to study the effects of chronic liver diseases on BBB permeability. However, contradictory changes in the BBB permeability to both small and large molecular weight markers have been reported in BDL rats (Bosoi et al. 2012; Dhanda and Sandhir 2017; Quinn et al. 2014; Stewart et al. 1989; Wahler et al. 1993; Xu et al. 2016). For instance, whereas some studies have reported an increase in the BBB permeability to Evans blue (EB) in BDL rats at five days (Quinn et al. 2014) or four weeks (Dhanda and Sandhir 2017) after surgery, others (Bosoi et al. 2012; Xu et al. 2016) have shown no change in the BBB permeability to this marker at 3, 7, or 14 days (Xu et al. 2016) or six weeks (Bosoi et al. 2012) following BDL. Similarly, both an increase (Dhanda and Sandhir 2017) and no change (Bosoi et al. 2012) in the BBB permeability to the low molecular weight marker sodium fluorescein (FL) have been reported in 4-week and 6-week BDL rats, respectively. Therefore, it is unclear how the BBB permeability to large and small molecular weight markers may change in this animal model of chronic liver disease.

Our relatively recent studies (Miah et al. 2015, 2017a; Shaik et al. 2013) on the effects of liver diseases on BBB permeability suggest that some of the contradictory results in the literature might be due to the methodological issues related to the method of calculation of BBB permeability changes, shortcomings of the markers, and/or analytical techniques used in these studies. A vast majority of the BBB permeability studies use the changes in the brain concentrations of the markers alone as a measure of alterations in the BBB permeability without consideration of possible changes in the systemic pharmacokinetics and plasma or blood concentrations of the markers. For example, with some exceptions (Wahler et al. 1993), the vast majority of the reported BBB permeability studies in BDL rats are based only on measuring the concentrations or activity of the markers in the brain alone (Bosoi et al. 2012; Dhanda and Sandhir 2017; Quinn et al. 2014; Stewart et al. 1989; Xu et al. 2016). Additionally, the changes in the brain concentrations of markers bound to proteins, such as FL (Miah et al. 2015) or EB (Saunders et al. 2015), may be simply due to a change in the protein binding of these markers in different disease states. Lastly, non-specific analytical methods, such as radioactive or fluorometric methods, may contribute to the contradictory results observed in the literature (Miah et al. 2017a).

We have developed a specific method for measuring the BBB permeability to low MW markers using a stable isotope labeled [^13^C]sucrose (Miah et al. 2016). The method is based on analyzing the marker in both plasma and brain by a specific LC-MS/MS method. As opposed to the widely-used low MW permeability marker FL, sucrose does not undergo binding to plasma proteins and is not subject to any known BBB transporters (Alqahtani et al. 2018; Palestine and Brubaker 1982; Sun et al. 2001). In this study, we used brain uptake clearance of [^13^C]sucrose, which uses both the brain concentrations and blood or plasma exposure of the marker, to investigate the time course of the effects of cholestatic liver disease on BBB permeability to low MW compounds in BDL rats.

## Materials and methods

### Chemicals and reagents

[UL-^13^C_12_]Sucrose (all the carbons in both glucose and fructose molecules are labeled with ^13^C isotope; denoted [^13^C]sucrose) and the internal standard, which was [UL-^13^C_6_^fru^]sucrose (all the carbons in the fructose molecule are labeled with ^13^C isotope), were purchased from Omicron Biochemicals (South Bend, IN, USA). Cytochrome c, 7-ethoxycoumarin, and 7-hydroxycoumarin were purchased from Sigma-Aldrich (St. Louis, MO, USA). LC-MS grade acetonitrile and water were procured from Fisher Scientific (Hampton, NH, USA). Ketamine and xylazine solutions were purchased from Lloyd Laboratories (Shenandoah, IA, USA). Heparin solution was purchased from APP Pharmaceuticals (Schaumburg, IL, USA). All the other reagents and chemicals were of high purity and purchased from commercially available sources.

### Animals and bile duct ligation surgery

Adult (255–330 g), male Sprague-Dawley rats were purchased from Charles River Laboratory (Wilmington, MA) and acclimated in single, ventilated cages with 12-h dark-light cycles in a temperature- and humidity-controlled room with free access to food and water. All the animal procedures used in this study were approved by Texas Tech University Health Sciences Center’s Institutional Animal Care and Use Committee and were consistent with the guidelines set by the Guide for the Care and Use of Laboratory Animals (National Research Council 2011). Animals were randomly assigned to two groups of bile duct ligation (BDL) and sham surgery (Sham), which were further divided into three groups studied five days, two weeks, or four weeks after the surgery, resulting in a total of six groups (*n* = 6–7/group).

For bile duct ligation, overnight-fasted rats were anesthetized with ketamine: xylazine (80:8 mg/kg) via intramuscular injection. After opening the abdomen, the common bile duct was doubly ligated close to the liver using two sutures and severed between the ligatures (Klein et al. 2017). Sham animals underwent laparotomy and manipulation of the bile duct without its ligation or resection. We used ketamine:xylazine because we recently (Noorani et al. 2023) showed that isoflurane anesthesia causes an opening of the BBB and increases the BBB permeability to sucrose and mannitol by a factor of two. However, ketamine:xylazine anesthesia does not have an impact on BBB permeability.

### Dosing and sampling

Sham and BDL rats were anesthetized with ketamine: xylazine, and a catheter was placed in their femoral artery for blood sample collection. A single dose (10 mg/kg) of [^13^C]sucrose was administered through the penile vein, and serial blood samples (~ 0.2 mL) were collected before and at 1, 5, 10, 15, 20, and 30 min following the administration of the marker. A portion of blood was centrifuged for plasma collection, and another portion was used to measure blood concentrations of [^13^C]sucrose. At the end of sampling, a catheter was placed in the left ventricle of the heart, and the whole body was perfused with ice-cold saline at a rate of 25 mL/min for 5 min, and the brain and liver were collected and snap-frozen in cold iso-pentane. All the samples were kept at –80°C before analysis.

### Analysis of [^13^C]sucrose in blood, plasma, and brain

Before analysis, plasma and blood samples were diluted 100- and 50-fold, respectively, with water, and the brain was homogenized in ice-cold water at a ratio of 1:9 to obtain brain homogenate. The concentrations of [^13^C]sucrose in the biological samples were determined using a previously reported validated UPLC-MS/MS method (Miah et al. 2016). Briefly, diluted blood, plasma, or brain samples (20 µL) were subjected to protein precipitation by the addition of 180 µL of acetonitrile: water (80:20), which contained [UL-^13^C_6_^fru^]sucrose as internal standard. The chromatographic separation was performed using an Acquity BEH amide column (50 mm × 3 mm, 1.7 µm) and an isocratic mobile phase of acetonitrile: water: ammonium hydroxide (72:28:0.1, v/v), run a flow rate 0.2 mL/min. Column temperature was maintained at 45 °C. The multiple reaction monitoring was in negative mode, and the transitions for [^13^C]sucrose and internal standard were 353➔92 m/z and 347➔89 m/z, respectively.

### Serum biochemical markers

The serum biochemical markers for cholestasis were quantitated using commercial assays according to the manufacturer’s instructions. Assay kits for the spectroscopic measurement of total bilirubin (Teco Diagnostics; Anaheim, CA, USA), total bile acids (TBA) (Crystal Chem; Elk Grove Village, IL, USA), and ammonia (Sigma-Aldrich; St. Louis, MO, USA) and fluorometric measurement of cholesterol (Cayman Chemical; Ann Arbor, MI, USA) were used.

### Liver microsome preparations and cytochrome P450 metabolism studies

Liver microsomes were prepared using standard ultracentrifugation methods (Lake 1987) and, after suspension in sucrose (250 mM), were stored at –80 °C. Total protein content was measured using the Bradford assay kit (Thermo Fisher Scientific, Waltham, MA). Carbon monoxide-bound total P450 contents were determined by Omura and Sato's method (Lake 1987). Cytochrome P450 reductase (CPR) activity was measured by the reduction of cytochrome c using a kinetic assay, as described before (Guengerich 1994). Ethoxycoumarin-O-dealkylation (ECOD) activity of the microsomes, as a general marker of the P450 activity (Waxman and Chang 2006), was determined by the conversion of ethoxycoumarin to hydroxycoumarin and LC-MS/MS measurement of the metabolite, as described in detail before (Chandrashekar et al. 2023).

### Pharmacokinetics analysis

The area under the plasma (${AUC}_{0-30}^{plasma}$) or blood (${AUC}_{0-30}^{blood}$) concentration-time curve from time zero to the last sampling time (30 min) was calculated by log trapezoidal rule using PKanalix software in the MonolixSuite (SimulationsPlus; Lancaster, CA, USA). To determine the BBB permeability to [^13^C]sucrose, apparent brain uptake clearance (*K*_*in*_) values were estimated based on both plasma (*K*_*in-plasma*_) and blood (*K*_*in-blood*_) AUC data (Bickel 2022):

1 $${K}_{in-blood}=\frac{{C}_{30}^{br}}{{AUC}_{0-30}^{blood}}$$

2 $${K}_{in-plasma}=\frac{{C}_{30}^{br}}{{AUC}_{0-30}^{plasma}}$$

where ${C}_{30}^{br}$ is the terminal brain concentration of the marker after the in-situ removal of the residual blood in the brain.

### Statistical analysis

Statistical analysis of data was performed using Prism software (GraphPad Software, LaJolla, CA). Two-way ANOVA, followed by Bonferroni multiple comparisons, was used for the comparison of biochemical markers, P450 metabolism parameters, AUC values, brain concentrations, and *K*_*in*_ values for Sham and BDL animals at three different time points of five days, two weeks, and four weeks. The relationships between the brain concentration or *K*_*in*_ and biochemical parameters were analyzed using linear regression analysis. In all cases, a *p* value < 0.05 was considered significant. Data are presented as mean ± SD or individual values. All the data were included in the analysis without any attempts at statistical elimination of potential outliers.

## Results

### Plasma biochemical markers

**Fig. 1:**
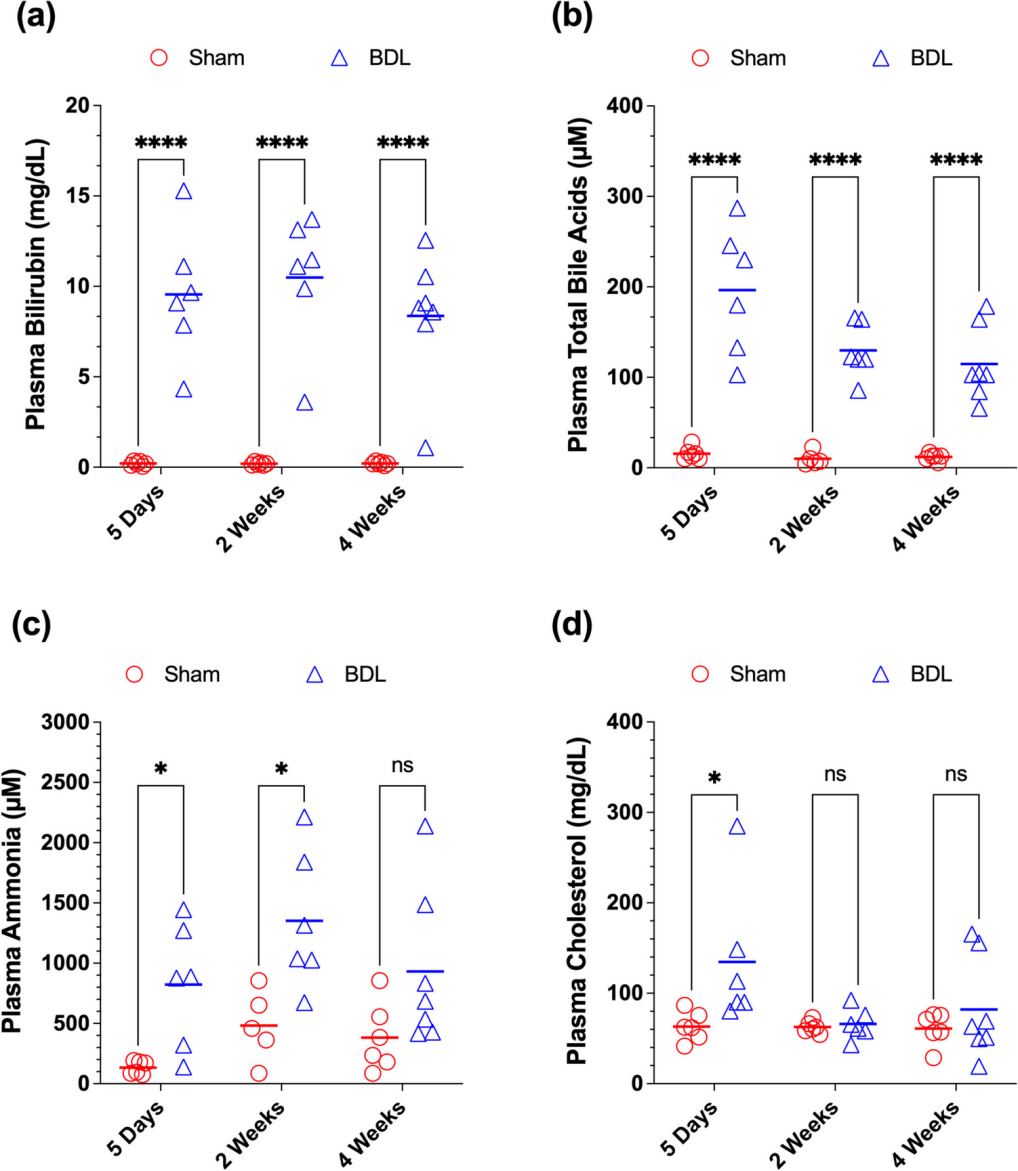
Plasma concentrations of biomarkers of cholestasis, total bilirubin (**a**), TBA (**b**), ammonia (**c**), and cholesterol (**d**), in the bile-duct ligated (BDL) and sham-operated (Sham) animals. Different groups of animals (*n* = 6–7/group) were subjected to the BDL or sham surgery, and plasma concentrations of the biomarkers were measured five days, two weeks, or four weeks after the surgery. Symbols and horizontal lines represent individual and mean values, respectively. Statistical significance (*, *p* < 0.05; ****, *p* < 0.0001, and ns, not significant) is based on two-way ANOVA, followed by Bonferroni multiple comparisons of the means

The plasma levels of cholestasis biomarkers are presented in Fig. 1. The plasma bilirubin concentrations in BDL animals were substantially (40-50 fold) higher (*p* < 0.0001) than their corresponding Sham animals for all the three time points of 5 Days, 2 Weeks, and 4 Weeks after the surgery (Fig. 1a). Similarly, the plasma total bile acids (TBA) concentrations in BDL animals were significantly (*p* < 0.0001) higher (10-13 fold) than those in Sham animals (Fig. 1b). However, the two-way ANOVA revealed that in addition to the differences between the Sham and BDL groups (*p* < 0.0001), the time after the surgery also had a significant effect (*p* < 0.05) on the results, with a trend toward lower TBA concentrations as the time of surgery progressed from 5 days to 4 weeks (Fig. 1b). The plasma ammonia levels in BDL animals were also higher (2.4–6.6 fold) than those in their corresponding Sham animals in all three time groups but the 2.4-fold difference for the 4 Week group did not reach statistical significance (*p* > 0.05), most likely due to the high variability in the data (Fig. 1c). For plasma cholesterol concentrations, there was only a significant (*p* < 0.05) increase (2.1 fold) in 5 Days BDL rats with no differences between BDL and Sham groups at the other two time points (Fig. 1d).

### Liver P450 content and activity

**Fig. 2:**
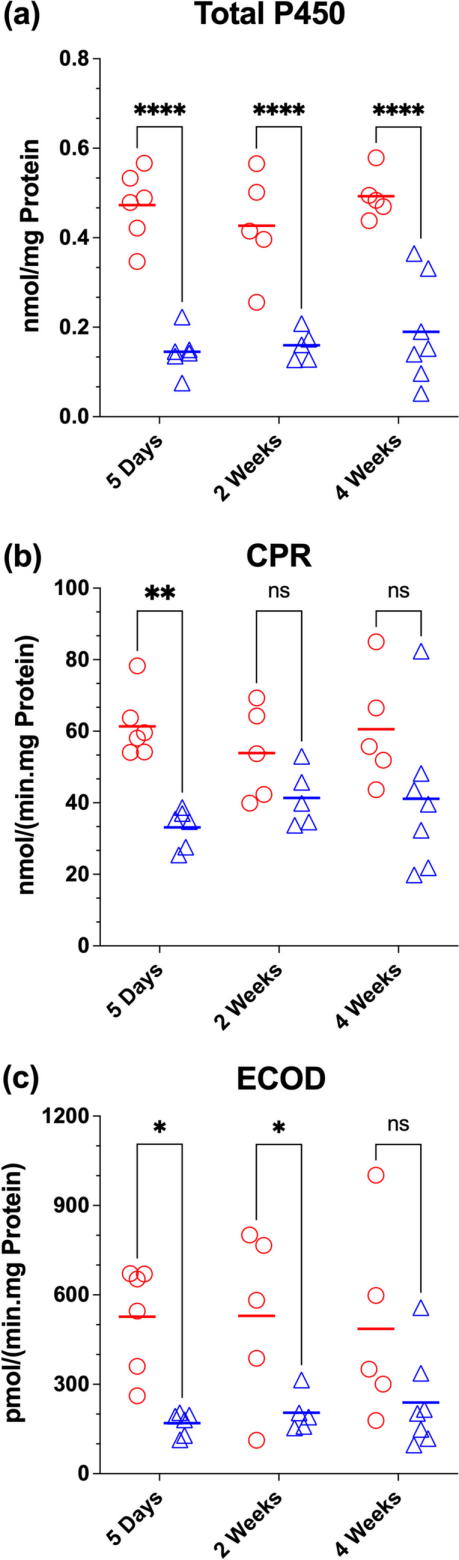
Liver microsomal total cytochrome P450 content (**a**), cytochrome P450 reductase (CPR) activity (**b**), and ethoxycoumarin-O-dealkylation (ECOD) activity (**c**) in the bile-duct ligated (BDL) and sham-operated (Sham) animals. Different groups of animals (*n* = 6–7/group) were subjected to the BDL or sham surgery, and liver microsomes were prepared five days, two weeks, or four weeks after the surgery. Symbols and horizontal lines represent individual and mean values, respectively. Statistical significance (*, *p* < 0.05; **, *p* < 0.01; ****, *p* < 0.0001; and ns, not significant) is based on two-way ANOVA, followed by Bonferroni multiple comparisons of the means

The total cytochrome P450 contents (Fig. 2a), CPR activities (Fig. 2b), and ECOD activities (Fig. 2c) for BDL and Sham groups at different time points after the surgery are presented in Fig. 2. The total cytochrome P450 content was significantly (*p* < 0.0001) depressed (60–70% decline) in BDL animals, compared with their corresponding Sham counterparts, at all the studied time points (Fig. 2a). The CPR activities in BDL animals were also lower than those in Sham animals but the decline (46%) was significant (*p* < 0.01) only for the 5 Days group (Fig. 2b). Similarly, the ECOD activities in BDL groups were lower than those in Sham animals. However, the decrease was significant (*p* < 0.05) only for the 5 Days (68% decline) and the 2 Weeks (61% decline) groups (Fig. 2c).

### Pharmacokinetics and brain uptake of [^13^C]sucrose

**Fig. 3:**
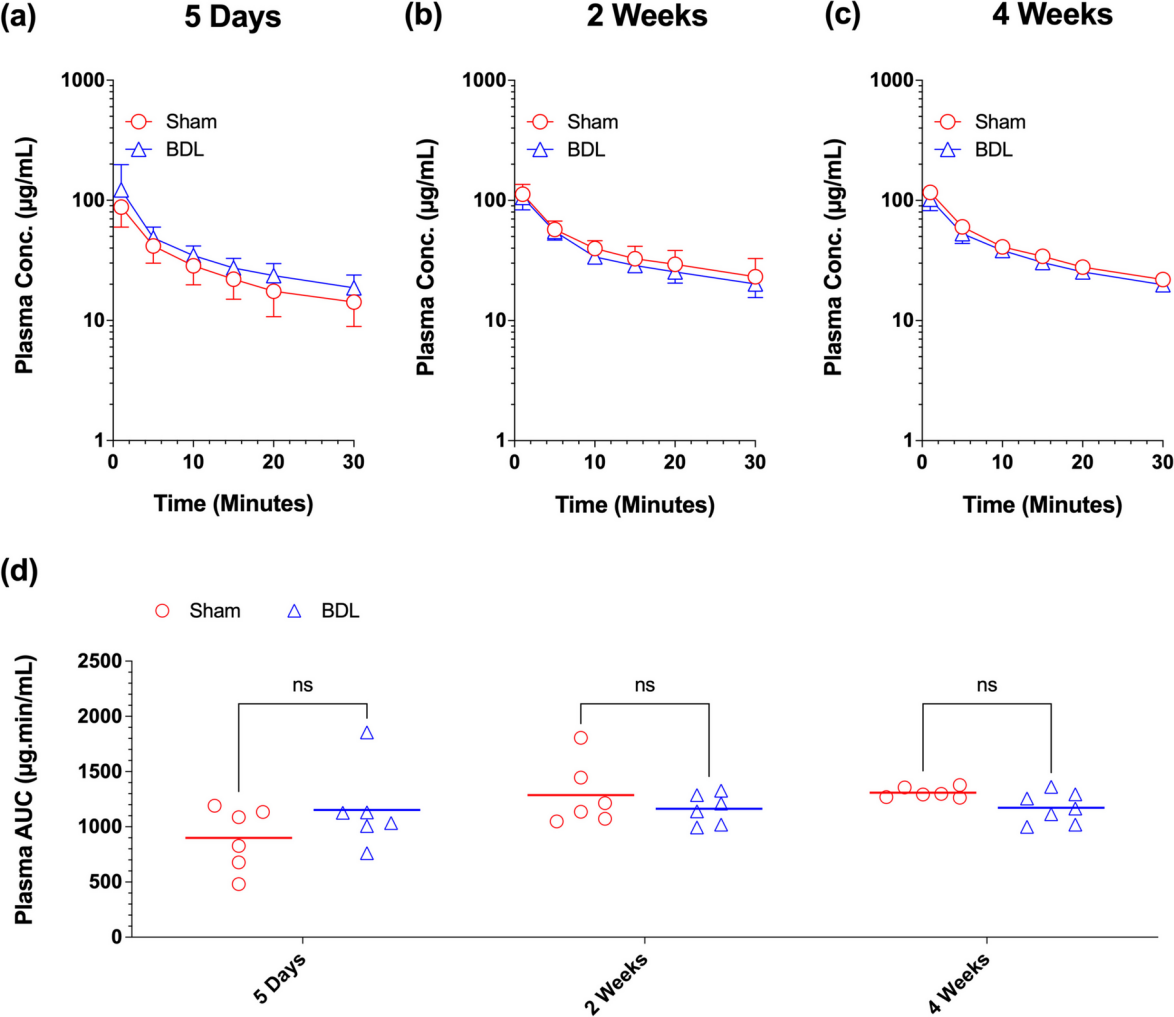
Plasma concentration-time courses (**a**-**c**) and AUC values (**d**) of [^13^C]sucrose in the bile-duct ligated (BDL) and sham-operated (Sham) animals. Animals were subjected to the BDL or sham surgery. Five days (**a**), two weeks (**b**), or four weeks (**c**) after the surgery, a single intravenous dose of [^13^C]sucrose (10/mg/kg) was administered to different groups (*n* = 6–7/group) of animals, and the plasma [^13^C]sucrose concentrations were measured. For the plasma concentration-time courses (**a**-**c**), symbols and bars represent the mean and SD, respectively. For the plasma AUC values, symbols and horizontal lines represent individual and mean values, respectively. Statistical significance (ns, not significant) is based on two-way ANOVA, followed by Bonferroni multiple comparisons of the means

The plasma concentration–time profiles of [^13^C]sucrose for the BDL and Sham groups after five days, two weeks, and four weeks of surgery, along with their respective AUC values, are presented in Fig. 3. As shown in the figure (Fig. 3a-c), the plasma concentrations of [^13^C]sucrose in BDL and Sham animals were almost superimposable at all three studied time points, resulting in similar ${AUC}_{0-30}^{plasma}$ values for the two surgical groups (Fig. 3d). Additionally, the corresponding plots based on the blood concentrations of [^13^C]sucrose are also presented in the Supplementary Data (Fig. S1). The blood data (Fig. S1) were consistent with the patterns observed with the plasma data (Fig. 3), indicating no significant differences between the ${AUC}_{0-30}^{blood}$ values for the two surgical groups (Fig. S1d). Neither the surgery (BDL and Sham) nor the time of the study affected the ${AUC}_{0-30}^{blood}:{AUC}_{0-30}^{plasma}$ ratios, which remained constant at 0.55–0.58 across all the groups. These AUC ratios are suggestive of hematocrit values of 0.42–0.45, considering that sucrose does not distribute to red blood cells.

**Fig. 4:**
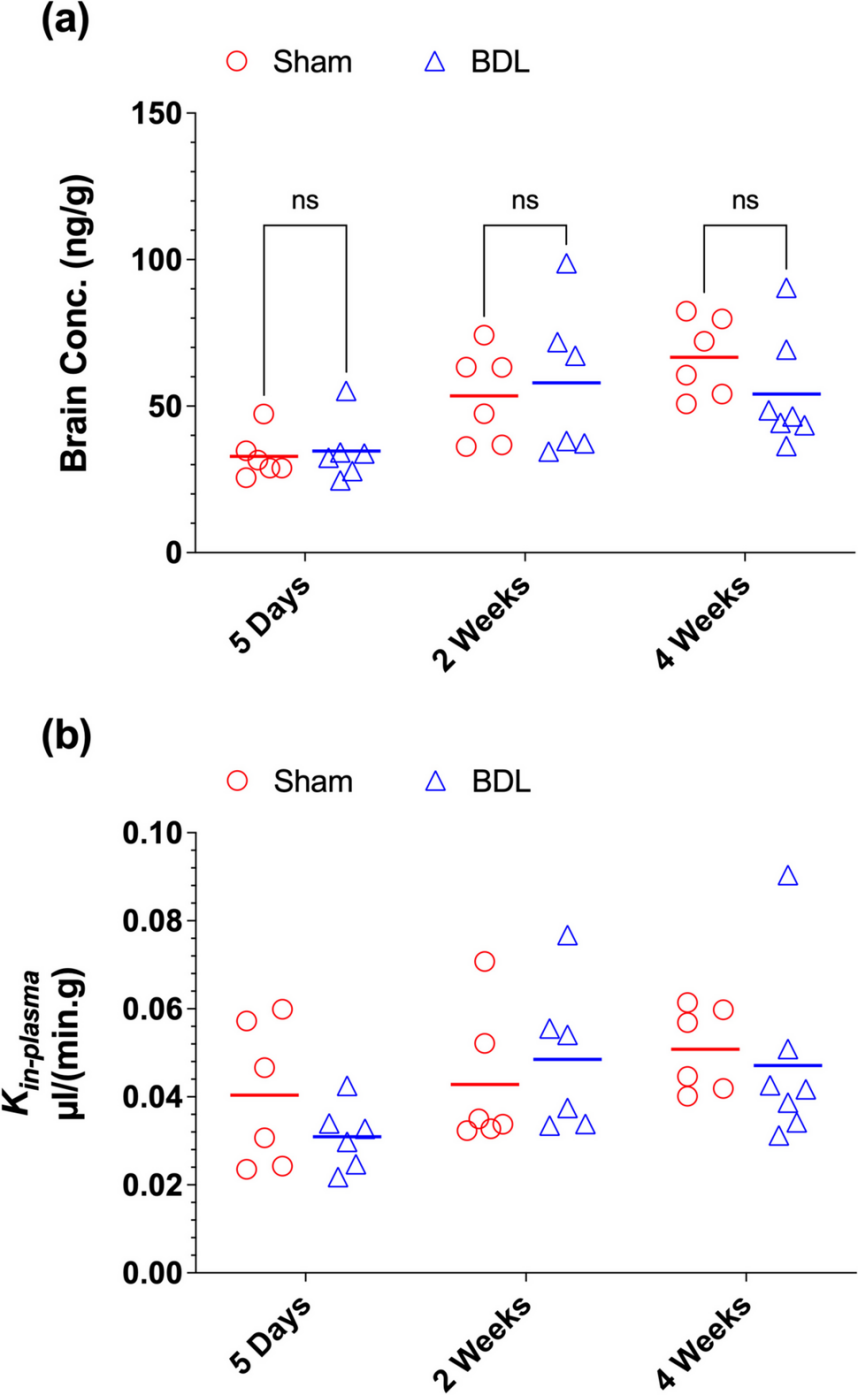
Terminal brain concentrations (**a**) and brain uptake clearance (*K*_*in-plasma*_) values (**b**) of [^13^C]sucrose in the bile-duct ligated (BDL) and sham-operated (Sham) animals. Animals were subjected to the BDL or sham surgery. Five days (**a**), two weeks (**b**), or four weeks (**c**) after the surgery, a single intravenous dose of [^13^C]sucrose (10/mg/kg) was administered to different groups (*n* = 6–7/group) of animals, and terminal (30 min) brain samples were collected. Symbols and horizontal lines represent individual and mean values, respectively. Statistical significance (ns, not significant) is based on two-way ANOVA, followed by Bonferroni multiple comparisons of the means

**Fig. 5:**
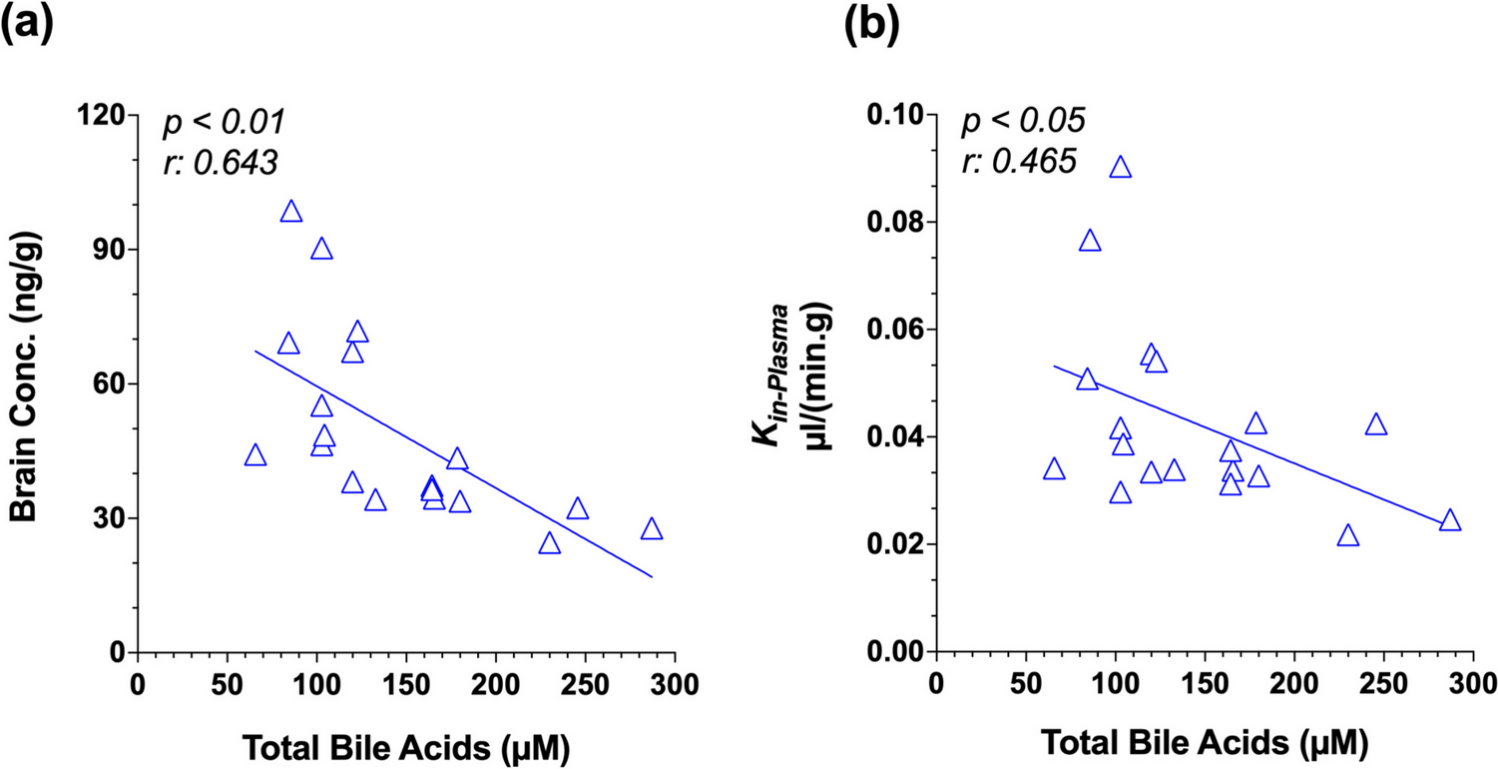
The relationship between the terminal brain concentration (**a**) or brain uptake clearance (**b**) of [^13^C]sucrose and plasma total bile acid concentrations in bile-duct ligated (BDL) animals. The analysis includes all the BDL animals studied five days, two weeks, or four weeks after the surgery (*n* = 6–7/group). Symbols and lines represent individual animals and the regression line, respectively. Statistical significance is based on linear regression analysis

The brain concentrations and uptake clearance (*K*_*in-plasma*_) values of [^13^C]sucrose are presented in Fig. 4. BDL did not significantly affect the brain concentrations (Fig. 4a) or the *K*_*in-plasma*_ values (Fig. 4b) of [^13^C]sucrose at any of the studied time points. As expected, the brain concentrations of [^13^C]sucrose (ng/g range) were negligible (Fig. 4a) relative to the substantially higher concentrations of the marker in the plasma (µg/mL range) (Fig. 3a-c). Similarly, the *K*_*in-plasma*_ values of [^13^C]sucrose in both BDL and Sham animals were very low (Fig. 4b). Analysis of the relationship between the brain concentration or *K*_*in-plasma*_ values of [^13^C]sucrose and the serum biochemical markers revealed a significant relationship only with the serum TBA concentrations in the BDL animals (Fig. 5). With an increase in the serum TBA concentrations, there was a decline in both the brain concentrations (*p* < 0.01) (Fig. 5a) and *K*_*in-plasma*_ values (*p* < 0.05) (Fig. 5b) of [^13^C]sucrose. Similar results were found when the blood, instead of serum, *K*_*in*_ values (*K*_*in-blood*_) were compared across different groups (Fig. S2a) or were analyzed against the serum TBA concentrations (Fig. S2b).

## Discussion

The normal BBB consists of tight junctions that strictly prevent the paracellular passage of small and large molecules and ions (Daneman and Prat 2015). However, under different pathophysiologic conditions, the integrity of BBB may be compromised, leading to the passage of endogenous and exogenous compounds and toxins and potential CNS adverse events. Liver diseases are associated with pathophysiological changes that increase the circulating concentrations of neuroinflammatory and neurotoxic compounds such as ammonia, bilirubin, lipopolysaccharides, and cytokines (Butterworth 2013; Claeys et al. 2021; Ott and Vilstrup 2014; Tranah et al. 2013). Therefore, there is a significant interest in the effects of liver diseases on the BBB barrier permeability (Butterworth 2016; Chastre et al. 2013; Cui et al. 2013; Nguyen 2012; Wang et al. 2011; Zaki et al. 1984). In the current study, we used sucrose as a BBB permeability marker in BDL rats to investigate the effects of cholestatic liver disease on BBB. Our results clearly show that the BBB permeability to sucrose, measured by the brain uptake clearance of the marker, remains unchanged at five days, two weeks, and four weeks after the surgery (Fig. 4c).

Other studies have reported increased (Dhanda and Sandhir 2017; Quinn et al. 2014), no change (Bosoi et al. 2012; Xu et al. 2016), or decreased (Wahler et al. 1993) BBB permeability in BDL rats. Most of these studies (Bosoi et al. 2012; Dhanda and Sandhir 2017; Quinn et al. 2014; Xu et al. 2016) used EB and/or FL as markers of BBB permeability. Although EB and FL are the top two most widely used markers in BBB permeability literature (Saunders et al. 2015), their use is associated with some methodological problems, which may be responsible, at least in part, for some of the observed discrepancies in the literature.

The use of EB (MW, 961) as a BBB marker is based on the assumption that after in vivo administration, the dye is completely bound to albumin, and EB-albumin cannot cross the intact BBB. However, it has long been reported (Allen and Orahovats 1950) that the binding of EB to albumin depends on the EB: albumin molar ratio. Indeed, in vivo doses routinely used in rodents produce measurable free EB concentrations in the plasma (Moos and Mollgard 1993). Additionally, EB's stability seems to depend on the injection vehicle (Saunders et al. 2015). Therefore, the use of EB as a marker of BBB permeability has been seriously questioned (Saunders et al. 2015). Using EB as a marker, Bosoi et al. (2012) reported no change in the BBB permeability six weeks after BDL surgery in rats. Similarly, Xu et al. (2016) showed no change in the BBB permeability to EB one or two weeks after the BDL surgery in rats. In contrast, increases in BBB permeability to EB in BDL rats have been reported five days (Quinn et al. 2014) or four weeks (Dhanda and Sandhir 2017) after the surgery. It is conceivable that these discrepancies might be due, at least in part, to confounding factors affecting the free fraction of the marker in plasma and its associated brain concentrations.

Sodium fluorescein is the most widely used small MW marker for BBB permeability studies in the literature (Saunders et al. 2015). Most investigators simply measure the concentrations of FL in the brain sometime after the in vivo administration of the marker as a measure of BBB permeability. Similar methods were also used to determine BBB permeability changes in BDL rats after the injection of FL, where both an increase (Dhanda and Sandhir 2017) and no change (Bosoi et al. 2012) were reported. We have previously shown that brain concentrations of FL were significantly increased after hepatectomy or hepatic ischemia-reperfusion injury (Miah et al. 2015). However, both free fraction of the marker in the plasma and free plasma AUC were also higher in the experimental groups than in control animals. When the brain concentrations were corrected for the plasma AUC of the unbound marker (i.e., free *K*_*in*_), there was no change in the BBB permeability to FL (Miah et al. 2015). Therefore, for accurate measurement of BBB permeability to FL, both its plasma free fraction and AUC should be used to correct the brain concentrations of the marker.

An ideal small MW marker for testing the integrity of the BBB tight junctions is expected to be polar (i.e., no transcellular transport), not bound to plasma proteins, and devoid of influx and efflux transporters at the BBB. Radioactive (^14^C) sucrose has been used as such a marker for more than half a century (Ferguson and Woodbury 1969). However, the use of radioactive sucrose is confounded by the presence of lipophilic impurities, which disproportionately accumulate in the brain, resulting in a 6-7-fold overestimation of true sucrose *K*_*in*_ (Miah et al. 2017b) and potentially inaccurate conclusions in the effects of pathophysiological conditions on the BBB permeability to sucrose (Miah et al. 2017a). Our data demonstrating that BDL in rats does not affect the BBB permeability (Fig. 4c) is based on the estimation of *K*_*in*_ of the isotope-labeled [^13^C]sucrose measured by the specific LC-MS/MS analysis (Miah et al. 2016), which is devoid of the potential methodological problems associated with [^14^C]sucrose and other low MW markers used in the BBB literature.

In addition to the most widely used small MW markers FL (MW, 376 Da) and sucrose (MW, 342 Da), mannitol with a MW of around half the MW of sucrose (182 Da) has also been used as a BBB permeability marker representing small molecules. In a recent study investigating the effects of anesthesia on BBB permeability (Noorani et al. 2023), we showed that although the absolute permeability of mannitol was about 2-fold higher than sucrose, the magnitude of the BBB opening under isoflurane/sevoflurane was the same for both sucrose and mannitol. Therefore, we expect that any small passive permeability marker in the low MW range > 180 Da would behave similarly to sucrose in BDL rats. For any small molecule toxin with more lipophilic character (subject to transcellular transport), potential changes in paracellular permeability are irrelevant.

Another potential area responsible for the discrepancy in the BBB permeability changes in liver diseases might be the correction method for the contaminating blood in the brain vasculature. A popular method is to wash out the blood in the brain vasculature using a physiological perfusate. However, depending on the rate and length of the perfusion, varying degrees of residual blood might be left in the brain tissue, which may affect the measured brain concentrations of the marker. One of the studies that reported no change in the BBB permeability to EB in BDL rats (Xu et al. 2016) perfused the animals with physiologic saline through the left ventricle until colorless perfusion fluid returned to the right atrium. However, the details of the perfusion, such as its rate and length, were not reported in other studies (Bosoi et al. 2012; Dhanda and Sandhir 2017; Quinn et al. 2014). The contribution of residual blood to total brain concentrations especially becomes significant after high doses and/or shorter times of marker circulation.

We selected a time course of five days to 4 weeks for our studies because the studies reporting an elevated BBB permeability were carried out between five days (Quinn et al. 2014) and four weeks (Dhanda and Sandhir 2017) after BDL surgery in rats. Additionally, a report (Kountouras et al. 1984) investigating the characteristics of the BDL model in Sprague-Dawley rats indicated that the ligation of the bile duct for 15 days or more resulted in fibrosis, with the majority of animals showing cirrhosis at four weeks.

The BDL model was based on double ligation plus bile duct sectioning between the two ligatures (Klein et al. 2017) to avoid potential bile duct re-epithelization and restoration of the bile flow. The success of the bile duct ligation was confirmed for each animal at the end of the study by laparotomy and visual inspection of the abdominal area, including the presence of a significant dilation of the bile duct anterior to the ligation, before collection of the liver for P450 studies.

The biochemical changes in the plasma of BDL rats observed in our study (Fig. 1) are consistent with the development of cholestasis reported in other studies in these animals (Bosoi et al. 2012; Dhanda and Sandhir 2017; Quinn et al. 2014; Xu et al. 2016). In terms of absolute plasma concentrations, our biochemical data in the Sham and BDL rats are within the range of previously reported values for bilirubin (Kountouras et al. 1984; Xu et al. 2016), total bile acids (Quinn et al. 2014; Xu et al. 2016), and cholesterol (Kinugasa et al. 1981). The absolute concentrations of ammonia in our studies (Fig. 1c) were higher than those previously reported in these animals (Espiritu-Ramirez et al. 2018; Tripathi et al. 2018), which might be due to in vitro generation of ammonia during the processing of plasma samples (Howanitz et al. 1984). Nevertheless, a potential overestimation of the absolute plasma concentrations of ammonia does not change the significant differences between the BDL and sham animals observed in our study (Fig. 1c).

Among the studied biomarkers, ANOVA revealed a significant (*p* < 0.05) effect of time after surgery on the TBA concentrations. Although the plasma TBA concentrations in BDL rats were significantly (*p* < 0.0001) higher than their corresponding Sham counterparts at all the time points, there was a decrease in the concentrations of TBA in the BDL rats at later times (Fig. 1b). Similar trends have also been observed in other studies (Quinn et al. 2014; Xu et al. 2016), indicating that TBA concentrations peak early after the surgery.

The significant negative correlation between brain concentrations or *K*_*in*_ values of [^13^C]sucrose and TBA concentrations in the serum, observed in our studies (Fig. 5 and S2b), suggests some protective effects of TBA on BBB. Earlier in vivo studies in rats using bile salts sodium dehydrocholate (Spigelman et al. 1983) or sodium deoxycholate (Greenwood et al. 1991) showed disruption of BBB and increased permeability of BBB markers after the injection of the bile salts. However, both studies used very high concentrations of bile salts. The study by Spigelman et al. (1983) showed consistent BBB disruption in all animals after injection of 1 mL solutions of 15% and 17.5% sodium dehydrocholate into the animals' carotid artery. These doses were also associated with significant toxicity, with most animals showing respiratory depression and convulsion soon after the injection, in addition to 16% mortality. Similarly, using an in situ rat brain perfusion model, Greenwood et al. (1991) reported disruption of BBB at very high perfusate concentrations of 1–1.5 mM of sodium deoxycholate. They attributed the BBB disruption to the lytic action of the bile acid, which was related to its detergent properties at high concentrations.

Other investigators have reported that the effects of bile acids on the BBB are more complex as they are dependent on both the concentrations (Greenwood et al. 1991) and degree of hydrophilicity (Palmela et al. 2015; Xing et al. 2023) of the bile salts. For example, whereas abnormally high concentrations (1–2 mM) of the hydrophobic bile salt deoxycholate damaged BBB, at lower concentrations (0.2 mM) of deoxycholate, BBB remained intact (Greenwood et al. 1991). Additionally, more recent investigations using in vitro models report a BBB protective effect for hydrophilic bile acids, such as ursodeoxycholic acid (UDCA) (Palmela et al. 2015). The latter study showed that UDCA prevents or restores the loss of BBB integrity induced by unconjugated bilirubin in monolayers of human brain microvascular endothelial cells. Additionally, a recent review (Xing et al. 2023) indicated that hydrophilic bile acids such as UDCA and its taurine conjugate have neuroprotective, anti-neuroinflammatory, anti-apoptotic, and antioxidative effects, which protect the BBB integrity. We are unaware of any in vivo investigation of the protective effects of bile salts on the BBB structure or permeability. However, our in vivo results, showing an association between a reduction in the *K*_*in*_ values and an increase in the TBA concentrations in the BDL group (Fig. 5b and Fig. S2b), agree with the protective effects of some bile acids reported using the in vitro models (Palmela et al. 2015). Further studies are needed to confirm the potential protective effects of bile acids on BBB integrity and their mechanisms using both in vitro and in vivo models.

In addition to the effects of BDL on the BBB permeability to sucrose, we also studied the changes in the P450-mediated metabolism in these animals. A potential change in the metabolism of xenobiotics in BDL animals is an important consideration when the BBB marker is subject to metabolism. In those cases, any changes in the brain concentrations of the marker, without correction for changes in its plasma concentrations, may not be indicative of changes in the BBB permeability. In the case of sucrose, any changes in the P450-mediated drug metabolism are not expected to affect the pharmacokinetics of the marker. Although it is well known that sucrose is metabolized in the gastrointestinal tract to glucose and fructose before absorption, intravenously administered sucrose is metabolically stable and is eliminated only by renal clearance of the unchanged marker (Deane et al. 1951).

The 60%-70% decline in the total P450 contents of the liver five days to four weeks after the BDL surgery (Fig. 2a) is in agreement with previous studies, which showed a 50% (Mackinnon and Simon 1974) or a 44% (Chen et al. 1995) decline, three or five days, respectively, after the BDL surgery in rats. Although not investigated in our current study, the effects of BDL on P450 enzymes are isoenzyme-specific with regard to both protein contents and activity (Kawase et al. 2019; Tateishi et al. 1998). Similarly, the 46% reduction in the CPR activity observed in our 5-day BDL animals (Fig. 2b) is almost identical to the 43% reduction reported in BDL rats five days after the surgery (Chen et al. 1995). Lastly, the 61%-68% decrease in the activity of ECOD in BDL rats (Fig. 2c) is in agreement with similar reductions in the total P450 content (Fig. 2a) and CPR activities (Fig. 2b). Overall, our data confirm previous reports that BDL in rats causes a substantial decline in the total P450 content and enzymatic activity.

Our previous study (Miah et al. 2017a) showed that using plasma or blood AUC to estimate *K*_*in*_ may result in different conclusions for disease-induced changes in BBB permeability to radioactive (^14^C) sucrose. Whereas the *K*_*in*_ values calculated based on the blood AUC showed a significant increase because of liver ischemia-reperfusion injury, when the *K*_*in*_ was estimated based on the plasma AUC, there was no difference in *K*_*in*_ between the sham and experimental groups. These discrepancies were attributed to small lipophilic impurities in [^14^C]sucrose. As opposed to [^14^C]sucrose, when [^13^C]sucrose was used, *K*_*in-Blood*_ or *K*_*in-Plasma*_ resulted in the same conclusion that the liver ischemia-reperfusion injury did not significantly affect the BBB permeability (Miah et al. 2017a). Our studies reported here (Fig. 4b and Fig. S2a) confirm that when [^13^C]sucrose is used, the measurement of the marker in blood or serum does not affect the study's outcome. Therefore, when using [^13^C]sucrose as a marker, investigators may quantitate the marker in either plasma or blood.

There was no difference between the Sham and BDL animals in their plasma (Fig. 3d) or blood (Fig. S1d) AUCs of [^13^C]sucrose. Because sucrose is not metabolized by the liver and is eliminated only through the kidneys by glomerular filtration, this suggests no change in the glomerular filtration in BDL rats.

It has been reported that the mean arterial blood pressure is reduced in BDL rats (Bomzon et al. 1990). Therefore, it may be argued that the delivery of [^13^C]sucrose in BDL rats is slower than the normotensive Sham animals. However, because of its extremely low permeability, BBB uptake clearance of sucrose is not flow-limited. Therefore, any potential change in the brain blood flow is not expected to affect the brain uptake clearance of sucrose.

One of the serious extrahepatic consequences of liver diseases is hepatic encephalopathy, which is a neurological complication manifesting itself as cognitive, psychiatric, and neurological disturbances ranging from mild symptoms to coma (Claeys et al. 2021; DeMorrow 2019). Bile duct ligated rats are an animal model of type C hepatic encephalopathy, which is associated with chronic liver diseases (DeMorrow et al. 2021). Although we did not conduct behavioral tests in our current study, several studies reviewed by DeMorrow et al. (2021) indicate that BDL rats develop signs of motor and learning deficits. However, the relationship between hepatic encephalopathy and changes in BBB permeability is not clear, as it is suggested that hepatic encephalopathy might occur in the absence of any BBB permeability changes (Butterworth 2013; Claeys et al. 2021).

Our study has a few limitations. For the plasma biomarkers, we focused on confirming the cholestasis in the BDL animals by measurement of bilirubin, total bile acids, and ammonia (Fig. 1). However, previous studies (Kountouras et al. 1984; Wu et al. 2018; Xu et al. 2016) have clearly shown that the BDL model also leads to liver injury, which manifests itself in increases the liver enzymes, such as aspartate (AST) and alanine (ALT) aminotransferases, and liver tissue histological abnormalities. During the review process, the authors were asked to conduct additional experiments to measure the plasma levels of AST and ALT and to provide a histological analysis of the liver structure to confirm the success of the bile duct ligation model for up to 4 weeks. We were not able to conduct these additional experiments because our plasma and liver samples were exhausted or deteriorated. Despite these limitations, the 40-fold higher (*p* < 0.0001) levels of bilirubin (Fig. 1a), 10-fold (*p* < 0.0001) higher levels of total bile acids (Fig. 1b), and 60% reduction (*p* < 0.0001) in the liver total P450 content (Fig. 2a) in BDL rats at 4 weeks coupled with the visual confirmation of the bile duct dilation at the end of the experiments are all suggestive of the success and preservation of bile duct ligation in our experiments.

Another study limitation is that we did not study the BBB structure, including tight junctions, using electron microscopy. However, previous studies (Nguyen et al. 2006; Nguyen 2010) in an acute liver failure model have shown an intact BBB structure with normal tight junctions even in the presence of an increased BBB permeability, measured by Evans blue. Therefore, a disruption of BBB integrity in our BDL model, which is less severe than acute liver failure, in the absence of any changes in the BBB permeability to sucrose is unlikely.

## Conclusions

In conclusion, the BBB permeability to the hydrophilic marker [^13^C]sucrose remained unchanged in bile duct-ligated rats up to four weeks after the surgery. This conclusion is based on the calculation of brain uptake clearance of the marker, which utilizes both the marker's brain and plasma (or blood) concentrations quantitated using a specific LC-MS/MS method.

## Supplementary Information

Below is the link to the electronic supplementary material.

Supplementary file1 (DOCX 547 KB)

## Data Availability

Except for the plasma or blood concentrations, all individual data are provided for each animal in this manuscript. Individual plasma or blood concentration-time data will be provided upon request from the corresponding author.
