# Supplementary material for: Bile duct ligation-induced cirrhosis does not alter the blood-brain barrier permeability to sucrose in rats
Source: Metab Brain Dis. 2024 Dec 5;40(1):53. doi: 10.1007/s11011-024-01486-6 (PMC11621172; doi:10.1007/s11011-024-01486-6)

**Supplementary File**

**Bile duct ligation-induced cirrhosis does not alter the blood-brain barrier permeability to sucrose in rats**

**Mohammad K. Miah^1,2^ . Ulrich Bickel^1,3^ . Reza Mehvar^1,4^**

^1^ Department of Pharmaceutical Sciences, Texas Tech University Health Sciences Center, Amarillo, Texas, USA

^2^ Present Address: Clinical Pharmacology & Quantitative Pharmacology, CPSS, AstraZeneca, Boston, Massachusetts, USA

^3^ Center for Blood-Brain Barrier Research, Texas Tech University Health Sciences Center, Amarillo, Texas, USA

^4^ Department of Biomedical and Pharmaceutical Sciences, School of Pharmacy, Chapman University, Irvine, California, USA

Corresponding author: Reza Mehvar, Department of Biomedical and Pharmaceutical Sciences, School of Pharmacy, Chapman University, 9401 Jeronimo Road, Irvine, California, USA; phone: 714.516.5490; E-mail: [mehvar@chapman.edu](mailto:mehvar@chapman.edu).

**Fig. S1.** Blood concentration-time courses (a-c) and AUC values (d) of [^13^C]sucrose in the bile-duct ligated (BDL) and sham-operated (Sham) animals. Animals were subjected to the BDL or sham surgery. Five days (a), two weeks (b), or four weeks (c) after the surgery, a single intravenous dose of [^13^C]sucrose (10/mg/kg) was administered to different groups (*n* = 6-7/group) of animals, and the blood [^13^C]sucrose concentrations were measured. For the blood concentration-time courses (a-c), symbols and bars represent the mean and SD, respectively. For the blood AUC values, symbols and horizontal lines represent individual and mean values, respectively. Statistical significance (ns, not significant) is based on two-way ANOVA, followed by Bonferroni multiple comparisons of the means.

**Fig. S2.** The brain uptake clearance of [^13^C]sucrose based on blood AUC (*K_in-blood_*) (a) and the relationship between *K_in-blood_* and plasma total bile acid concentrations in bile-duct ligated (BDL) animals. The analysis includes all the BDL animals studied five days, two weeks, or four weeks after the surgery (n = 6-7/group). Symbols and lines represent individual animals and the regression line, respectively. Statistical significance is based on linear regression analysis.

**Fig. S1**


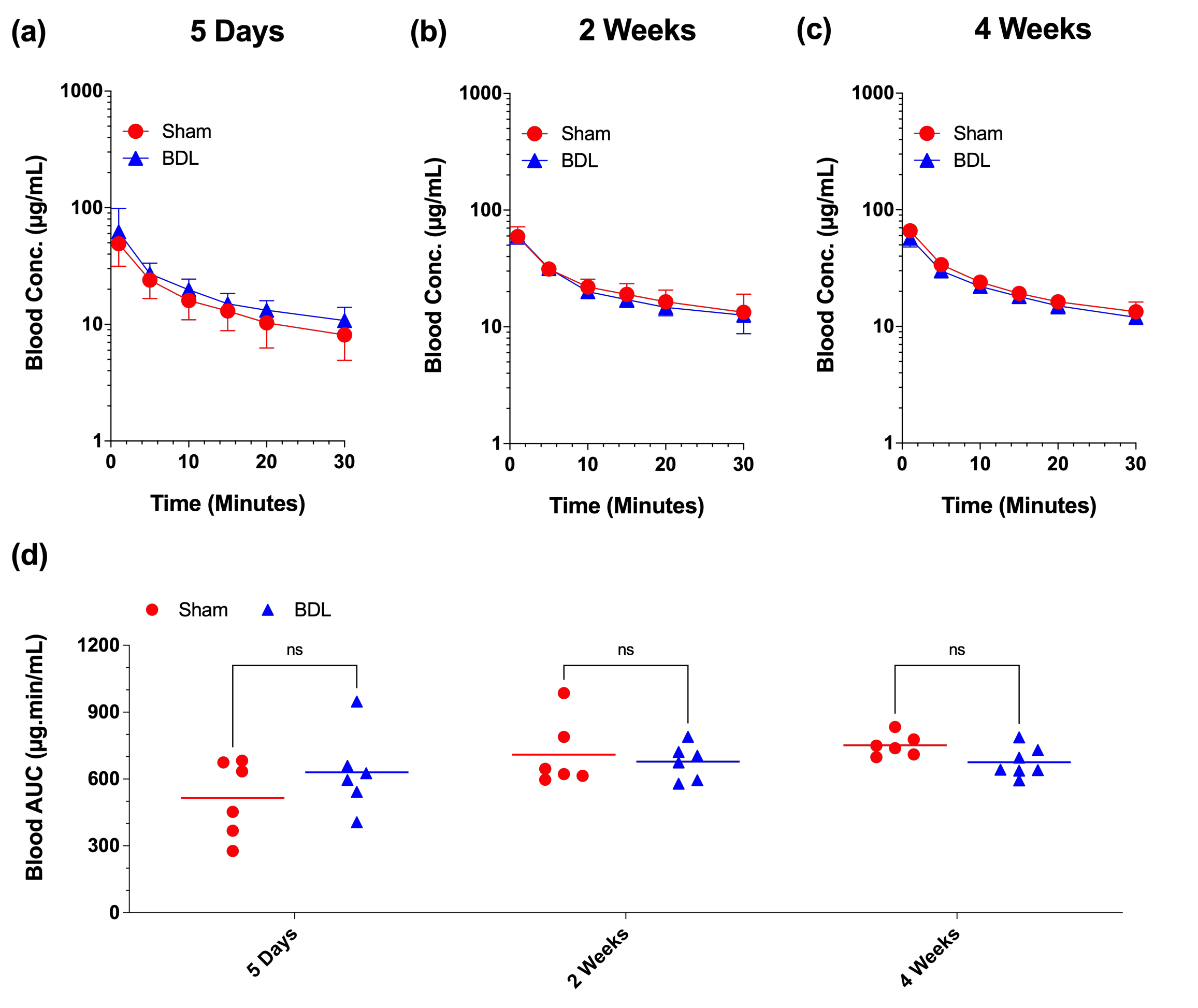


**Fig. S2**


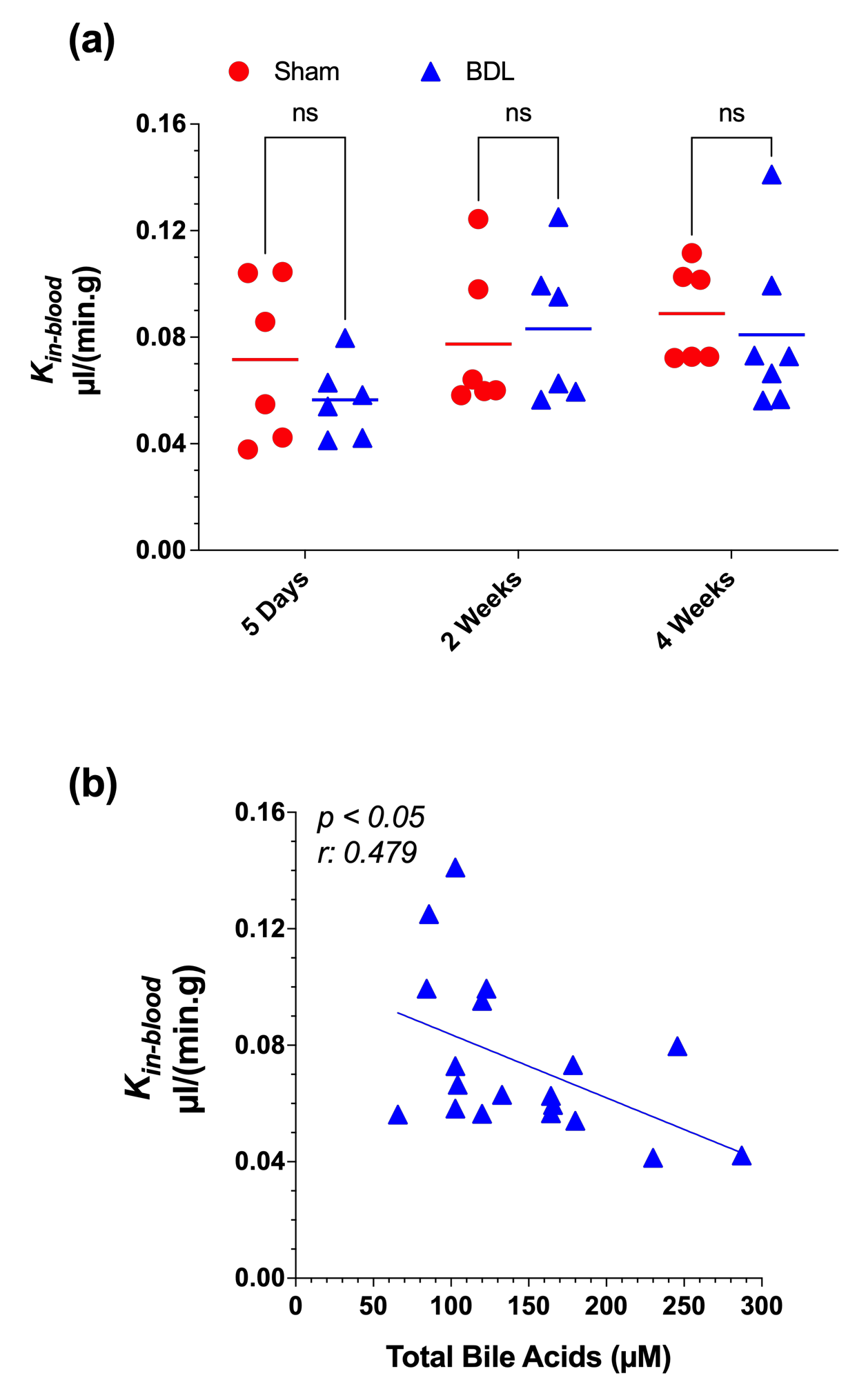

Supplement: Supplementary file 1 — Supplementary file1 (DOCX 547 KB) [file 11011_2024_1486_MOESM1_ESM.docx]
